# Swim Training Modulates Mouse Skeletal Muscle Energy Metabolism and Ameliorates Reduction in Grip Strength in a Mouse Model of Amyotrophic Lateral Sclerosis

**DOI:** 10.3390/ijms20020233

**Published:** 2019-01-09

**Authors:** Damian Jozef Flis, Katarzyna Dzik, Jan Jacek Kaczor, Karol Cieminski, Malgorzata Halon-Golabek, Jedrzej Antosiewicz, Mariusz Roman Wieckowski, Wieslaw Ziolkowski

**Affiliations:** 1 Department of Bioenergetics and Nutrition, Gdansk University of Physical Education and Sport, 80-336 Gdansk, Poland; damian.flis@gumed.edu.pl; 2 Department of Neurobiology of Muscle, Gdansk University of Physical Education and Sport, 80-336 Gdansk, Poland; kasi.dzik@gmail.com (K.D.); kaczorj@gumed.edu.pl (J.J.K.); 3 Department of Bioenergetics and Physiology of Exercise, Faculty of Health Sciences, Medical University of Gdansk, 80-211 Gdansk, Poland; jant@gumed.edu.pl; 4 School of Postgraduate Studies, Gdansk University of Physical Education and Sport, 80-336 Gdansk, Poland; karol.cieminski@awf.gda.pl; 5 Department of Physiotherapy, Faculty of Health Sciences, Medical University of Gdansk, 80-211 Gdansk, Poland; malgorzata.halon-golabek@gumed.edu.pl; 6 Department of Biochemistry, Gdansk University of Physical Education and Sport, 80-336 Gdansk, Poland; 7 Nencki Institute of Experimental Biology, 02-093 Warsaw, Poland; m.wieckowski@nencki.gov.pl

**Keywords:** neurodegeneration, mitochondria, ALS, oxidative stress, exercise, bioenergetics

## Abstract

Metabolic reprogramming in skeletal muscles in the human and animal models of amyotrophic lateral sclerosis (ALS) may be an important factor in the diseases progression. We hypothesized that swim training, a modulator of cellular metabolism via changes in muscle bioenergetics and oxidative stress, ameliorates the reduction in muscle strength in ALS mice. In this study, we used transgenic male mice with the G93A human SOD1 mutation B6SJL-Tg (SOD1^G93A^) 1Gur/J and wild type B6SJL (WT) mice. Mice were subjected to a grip strength test and isolated skeletal muscle mitochondria were used to perform high-resolution respirometry. Moreover, the activities of enzymes involved in the oxidative energy metabolism and total sulfhydryl groups (as an oxidative stress marker) were evaluated in skeletal muscle. ALS reduces muscle strength (−70% between 11 and 15 weeks, *p* < 0.05), modulates muscle metabolism through lowering citrate synthase (CS) (−30% vs. WT, *p* = 0.0007) and increasing cytochrome c oxidase and malate dehydrogenase activities, and elevates oxidative stress markers in skeletal muscle. Swim training slows the reduction in muscle strength (−5% between 11 and 15 weeks) and increases CS activity (+26% vs. ALS I, *p* = 0.0048). Our findings indicate that swim training is a modulator of skeletal muscle energy metabolism with concomitant improvement of skeletal muscle function in ALS mice.

## 1. Introduction

Amyotrophic lateral sclerosis (ALS) is an incurable, chronic neurodegenerative disease characterized by selective death of motoneurons in the motor cortex, brainstem, and spinal cord, which controls muscle action [1]. This disease is phenotypically characterized by loss of muscle tone, paresis, muscle atrophy, and spasticity [2]. These degenerative changes result in the patient’s loss of overall ability to initiate and control voluntary movements, with the exception of the eyes. Approximately 90% of ALS is a sporadic form of disease (sALS), for which the etiology is unknown, and the remaining percentage is a genetically determined form (fALS). In clinical terms, both forms are virtually identical. Since 1993, the study of the etiology of fALS has focused on mutations in the gene encoding superoxide dismutase type 1 (SOD1), which occurs in nearly one-fifth of all cases of familial form of the disease [3,4]. The discovery of the role of mutations in the *SOD1* gene in the etiology of ALS has contributed to constructing the most suitable animal test model of the disease (type fALS), containing the human *SOD1 G93A* transgene described by Gurney [5], where glycine is changed to alanine at position 93. The similarity in the course of ALS between the human and animal form allows the study of the pathomechanisms of the disease and testing the factors that may mitigate its course, or even extend the life span, in ALS animals. Notably, causes of neurodegeneration in ALS occur outside of the nervous system. Overexpression of hSOD1 G93A in skeletal muscle not only initiates motoneuron death [6], but also causes profound muscle atrophy [6,7]. Additional support for the view that muscle plays a key role in ALS pathogenesis has been provided by the observations that the destruction of neuromuscular junctions is linked to oxidative stress induced by tissue-specific breakdown of muscle mitochondria [8].

Identification of abnormalities in skeletal muscle at the early stage of ALS, preceding the onset of the disease, seems to be important for diagnosis and treatment of patients. Our previous study showed that skeletal muscle in rats bearing the *hSOD1 G93A* transgene already showed evidence of oxidative stress at the early stages of disease [9,10]. In addition, the ALS rats exhibited an impairment of insulin signaling and iron metabolism in the skeletal muscle that preceded muscle atrophy [11]. Other symptoms, observed in skeletal muscle, are characteristic for the early stage of disease including the upregulation of proteasome activity and autophagy activation [12], reduction of mechanical properties of gastrocnemius-soleus muscle group [13], the impairment of l-type Ca^(2+)^ channel—a key regulator of both mass and force of skeletal muscle [14]—loss of forelimb strength (grip test) [15], abnormal mitochondrial dynamics [16], and other morphological and functional mitochondria abnormalities [17].

Mitochondrial dysfunction and oxidative stress are associated with the development of neurodegenerative diseases [18,19,20,21,22,23]; however, the relationship between these factors and skeletal muscle strength dysfunction at the early stage of ALS is not completely understood.

Mitochondrial dysfunction has been observed in skeletal muscles of *hSOD1 G93A* mice, which are related to mutation in SOD1. SOD1 mutations are associated with misfolding and mislocalization of the SOD1 protein. While the normal SOD1 protein is usually found in the cytosol, mutant SOD1 accumulates within mitochondria and appears to contribute to many of the mitochondrial perturbations such as impaired adenosine triphosphate (ATP) production, altered calcium buffering, and initiation of apoptosis [24,25,26,27]. Luo et al. demonstrated that SOD1 G93A formed aggregates in mitochondria of skeletal muscle, and this aggregation led to mitochondrial depolarization, disruption of the mitochondrial network, and dynamics in skeletal muscle [16] Moreover, dysfunctional mitochondria can be a source of reactive oxygen species (ROS) generation and cellular oxidative stress [28,29,30]. In addition, Xiao et al. showed an increased ROS generation prior to the onset of ALS symptoms (at the age of 2 months), whereas the onset of ALS symptoms (at the age of four months) was associated with drastic increase in ROS generation [31]. Decrease in oxidative stress seems to be one of the most important strategies for treating ALS disease and other neuromuscular disorders [32,33,34]. Regulation of metal homeostasis and decrease in oxidative stress, similar to the benefits of swim training, leads to the prolongation of the lifespan of transgenic mice [35,36]. Deforges et al. [15] documented that swim training delays the onset of the first symptoms of the disease, sustains the motor function, and increases the life span of ALS mice by about 25 days. Similar results were obtained in our recent studies [36]. In contrast to running exercise, large-scale motoneurons are activated by swimming. The swimming-based program is a high frequency exercise associated with high hindlimb movement amplitude (373.9 ± 47.6 cycles min^−1^ and 4.86 ± 0.40 cm), preferentially activating a sub-population of large motoneurons [37], which may play an important role in the protection of muscle fibers in ALS. Biochemical and physiological changes induced by exercise have been quite well defined. Data have shown that swimming exercise modifies mitochondria structure and function due to reduction of oxidative stress, improvement of mitochondrial bioenergetics, and regulation of the concentration of mitochondrial cholesterol [36,38,39].

We hypothesized that at the early stages of the disease, swim training is already a modulator of cellular metabolism via changing muscle bioenergetics, and modulating the oxidative stress can influence the reduction in muscle strength. We also proposed checking which of the parameters are correlated with the reduction in muscle strength in ALS mice, which may be used as potential biomarker of the early phase of the disease. Therefore, the aim of this study was to investigate the effects of swim training and ALS disease on mitochondrial bioenergetics, oxidative stress, and on changes in muscle strength of *hSOD1 G93A* mice at the early stage of the disease.

## 2. Results

**Figure 1:**
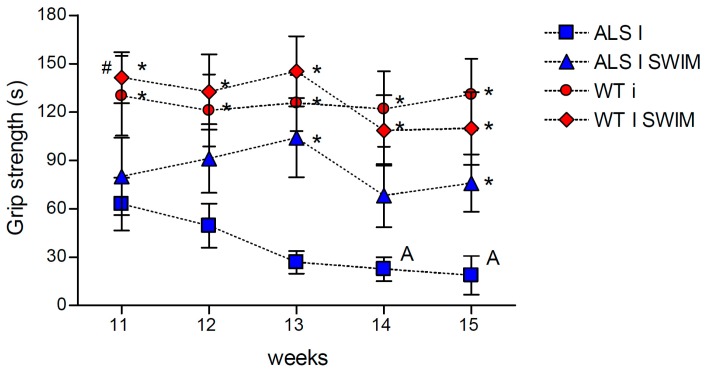
The effects of swim training on grip strength in the ALS mice. Swim training maintained grip strength in ALS mice. There were significant differences between the groups: * *p* < 0.05 vs. symptomatic ALS mice (ALS I), ^#^ *p* < 0.05 vs. ALS I SWIM; ^A^ *p* < 0.05, between the indicated time points and 11 week in ALS I group (LSD post hoc test). The data are presented as the means ± SEM (*n* = 8 in each group). (*n* = 8 for each group).

The progression of ALS leads to muscle weakness and atrophy, which are associated with deterioration in muscle strength. Therefore, to assess the effects of swim training on the grip strength of ALS mice, we subjected both wild-type (WT) and *hSOD1 G93A* mouse groups to a regimen of exercise. Over time, untrained mice lost grip strength as a result of the progression of the disease. According to the expectations, we observed that swim training maintained the grip strength in ALS mice (Figure 1).

**Figure 2:**
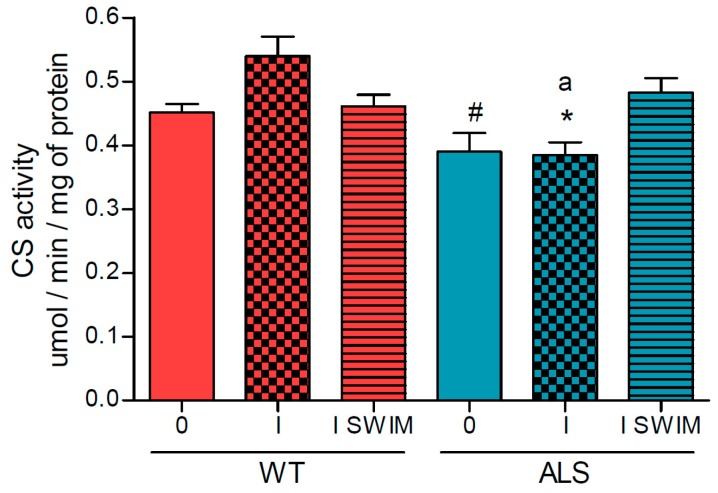
Citrate synthase activity in skeletal muscle of ALS and WT mice. Citrate synthase (CS) was measured in skeletal muscle homogenates. There were significant differences between the groups: * *p* = 0.0048, ^#^ *p* = 0.0075 vs. ALS I SWIM (LSD post hoc test); ^a^ *p* = 0.0007 between the indicated ALS and corresponding WT groups. The data are presented as the means ± SEM (*n* = 8 in each group).

As we postulated that the decline in the bioenergetics in muscles is a factor favoring the progression of the disease, we decided to measure the activities of individual enzymes or protein complexes involved in oxidative metabolism. The activity of citrate synthase (CS), the first enzyme in the tricarboxylic acid (TCA) cycle, was reduced in the ALS I group of mice (0.385 ± 0.02 μmol/min/mg of protein), compared with the corresponding WT mice (0.540 ± 0.03 μmol/min/mg of protein) (Figure 2). Additionally, there was a small association of swim training with an increase in CS activity (ALS I SWIM vs. ALS I) (*p* = 0.0048, LSD *post hoc* test) (Figure 2).

**Figure 3:**
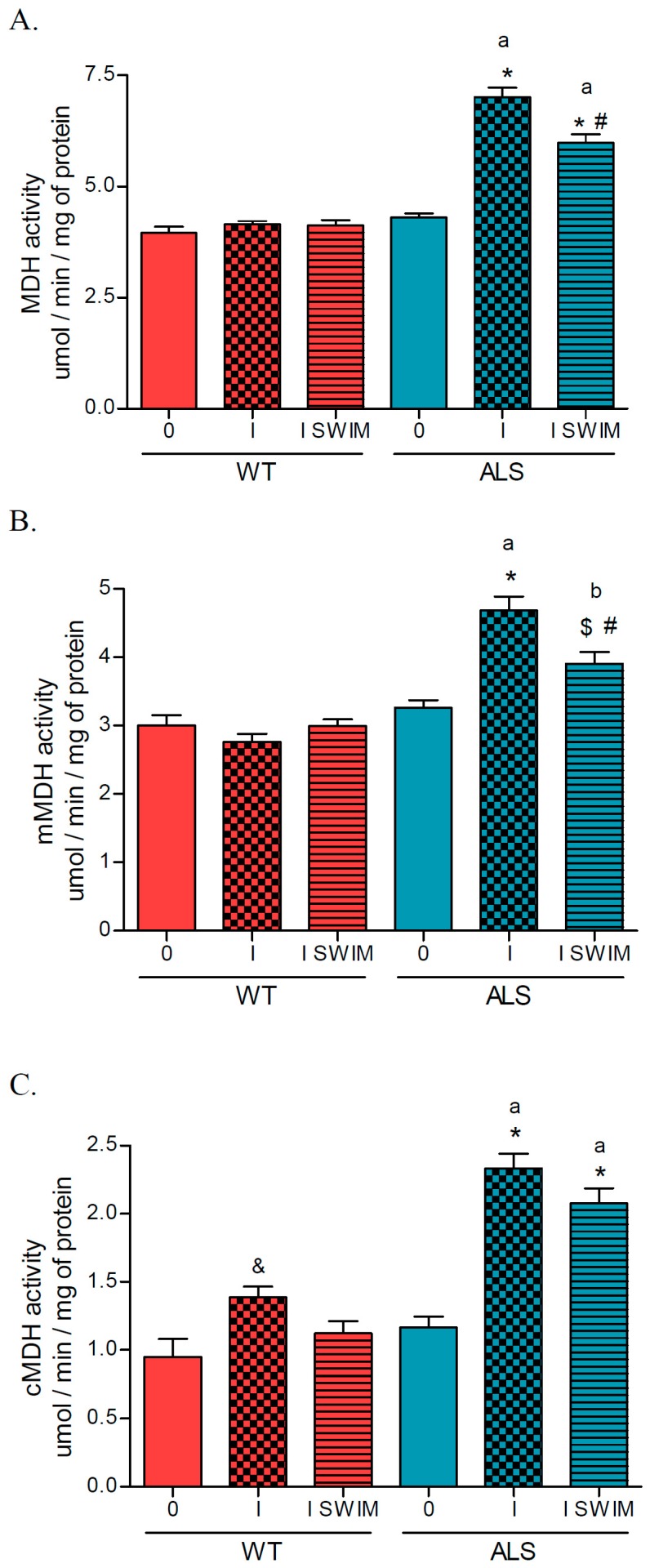
Malate dehydrogenase activity in skeletal muscle of ALS and WT mice. Total malate dehydrogenase (MDH) (**A**), mitochondrial malate dehydrogenase (mMDH) (**B**), and cytosolic malate dehydrogenase (cMDH) (**C**) were measured in skeletal muscle homogenates. There were significant differences between the groups: * *p* = 0.0001 vs. ALS 0, ^#^ *p* = 0.0054 vs. ALS I, ^$^ *p* = 0.0357 vs. ALS 0, ^&^ *p* = 0.04 vs. WT 0; ^a^ *p* = 0.000, ^b^ *p* = 0.0009 between the indicated ALS and corresponding WT groups. The data are presented as the means ± SEM (*n* = 8 in each group).

The activity of total malate dehydrogenase (MDH) increased in the ALS I and ALS I SWIM groups (7.018 ± 0.21 and 5.982 ± 0.19 μmol/min/mg of protein, respectively) vs. the ALS 0 group (4.300 ± 0.09 μmol/min/mg of protein) (*p* = 0.0001) and corresponding WT group of mice (*p* = 0.0001). However, swim training was associated with a decrease in MDH activity (ALS I SWIM vs. ALS I) (*p* = 0.0003). No changes in MDH enzyme activity were observed in the WT groups of mice (Figure 3A). The activity of mitochondrial malate dehydrogenase (mMDH) increased in the ALS I and ALS I SWIM groups (4.685 ± 0.20 and 3.904 ± 0.17 μmol/min/mg of protein, respectively) vs. the ALS 0 group (3.265 ± 0.11 μmol/min/mg of protein) and corresponding WT group of mice. However, there was a swim-training-associated decrease in mMDH activity (ALS I SWIM vs. ALS I) (*p* = 0.0054). No changes in mitochondrial MDH activity were observed in the WT groups of mice (Figure 3B). The activity of cMDH increased in the ALS I and ALS I SWIM groups (2.333 ± 0.11 and 2.077 ± 0.11 μmol/min/mg of protein, respectively) vs. the ALS 0 group (1.166 ± 0.08 μmol/min/mg of protein) (*p* = 0.0001) and corresponding WT group of mice (*p* = 0.0001). The activity of cMDH was higher in WT I vs. WT 0 group of mice (Figure 3C).

**Figure 4:**
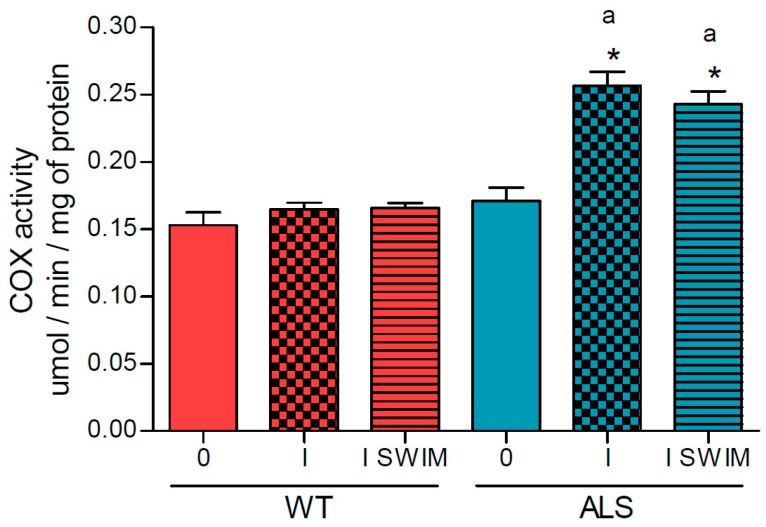
Cytochrome c oxidase activity in skeletal muscle of ALS and WT mice. The activity of COX was measured in skeletal muscle homogenates. There were significant differences between the groups: * *p* = 0.0001 vs. ALS 0; ^a^ *p* = 0.0001 between the indicated ALS and corresponding WT groups. The data are presented as the means ± SEM (*n* = 8 in each group).

Cytochrome c oxidase (COX) activity increased in the ALS I and ALS I SWIM groups (0.257 ± 0.01 and 0.243 ± 0.01 μmol/min/mg of protein, respectively) vs. the ALS 0 group (0.171 ± 0.01 μmol/min/mg of protein) (*p* = 0.0001) and corresponding WT group of mice (*p* = 0.0001). No changes in the activity of this enzyme were observed in the WT groups of mice (0.153 ± 0.01, 0.165 ± 0.01, and 0.166 ± 0.01 μmol/min/mg of protein in WT 0, WT I, and WT I SWIM, respectively) (Figure 4).

As MDH activity is correlated with COX activity (*R* = 0.84, *p* = 0.000002), we think that this is a compensatory response, whereby there are no alterations in mitochondrial respiration (Table 1). The grip strength was correlated only with the activity of CS (*R* = 0.68, *p* = 0.005).

**Figure 5:**
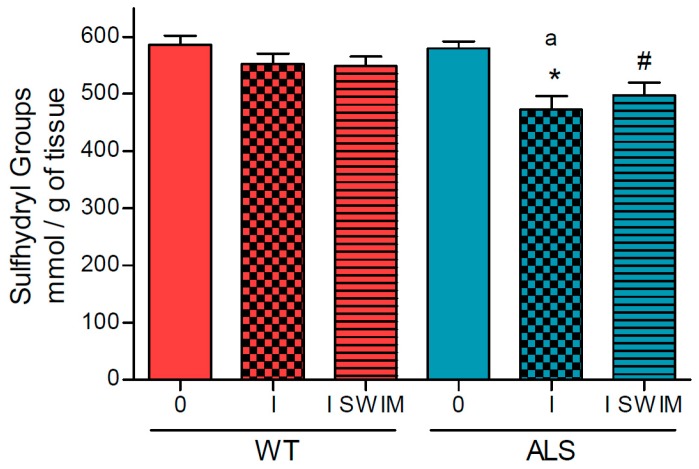
Oxidative stress parameters (sulfhydryl groups) in skeletal muscle homogenates of the ALS and WT mice. Sulfhydryl groups were measured in skeletal muscle homogenates. There were significant differences between the groups: * *p* = 0.0019 vs. ALS 0, ^#^ *p* = 0.03 vs. ALS 0; and ^a^ *p* = 0.038 between the indicated ALS and corresponding WT groups. The data are presented as the means ± SEM (*n* = 8 in each group).

Oxidative stress is another factor that accompanies the development of ALS. Therefore, we decided to measure the concentration of sulfhydryl (SH) groups in the skeletal muscle of ALS and WT mice. In symptomatic ALS mice (ALS I), we observed lower SH groups concentration compared with the ALS 0 group of mice (472.4 ± 23.96 and 579.9 ± 11.57 nmol/g of tissue, respectively) (*p* = 0.0019). The level of SH groups was also higher in ALS 0, as compared with the trained ALS mice (497.6 ± 21.92 nmol/g of tissue) (*p* = 0.0299) (Figure 5). There were no significant changes in the level of SH groups in any WT group of mice (586.0 ± 15.30, 552.2 ± 18.54 and 549.0 ± 16.02 nmol/g of tissue in WT 0, WT I, and WT I SWIM, respectively) (Figure 5). The concentration of SH groups was significantly correlated with COX activity (*R* = –0.79, *p* = 0.000003).

## 3. Discussion

**Figure 6:**
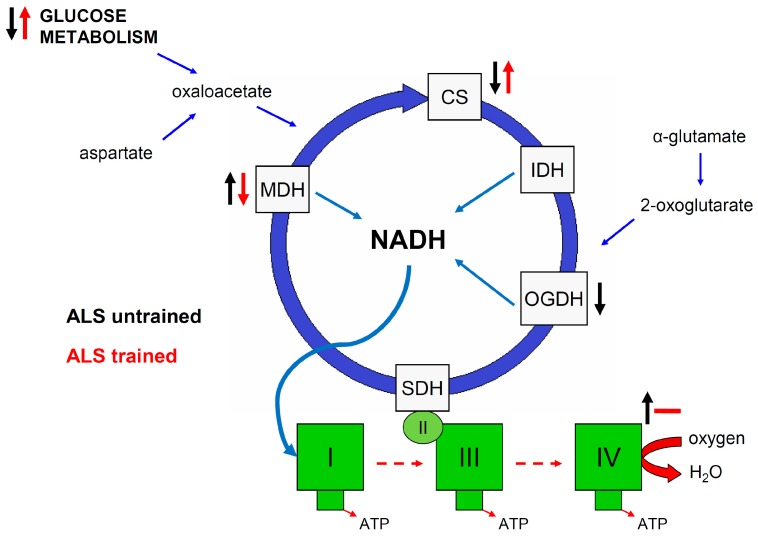
Skeletal muscle mitochondrial energy metabolism modulation in ALS untrained and ALS swim-trained mice, based on Desseille et al. [26], Tefera et al. [40] and our results. Black arrows indicate changes observed in skeletal muscle of ALS mice at symptomatic stage of disease vs. ALS 0 or WT. Red arrows indicate swim-training-induced changes in skeletal muscle of ALS mice at symptomatic stage of disease vs. untrained symptomatic ALS mice. ↑—increased; ↓—decreased; --—no changes. CS—citrate synthase, IDH—Isocitrate dehydrogenase, OGDH—2-oxoglutarate dehydrogenase, MDH—malate dehydrogenase, I, II, III, IV—complexes of mitochondrial electron transfer chain.

In this study, we demonstrated that at the symptomatic stage of ALS, the mouse skeletal muscle experiences increased oxidative stress, changes in energy metabolism enzyme activities, and a significant reduction in muscle strength compared to WT animals and ALS mice without disease symptoms. Swim training significantly decreased the decline in muscle strength in ALS animals. For the first time, we indicated that only CS activity is correlated with changes in muscle strength in ALS animals with the first symptoms of the disease (Figure 6).

In the group of ALS mice showing the first symptoms of the disease, we noticed the mutual relations between COX and MDH activity and the level of SH groups and between COX and MDH isoenzymes activities.

### 3.1. Effect of ALS on Energy Metabolism and Oxidative Stress in Symptomatic ALS Mice

Decreased energy metabolism is a characteristic sign of ALS disease [40]. Reduced activity of the TCA cycle, glucose metabolism, the level of TCA C4 intermediates and mitochondrial respiration, as well as ATP production by oxidative phosphorylation (OXPHOS) system lead to defects in skeletal muscle energy metabolism of ALS mice, and are some of the important factors of disease progression [40]. For this reason, we measured mitochondrial bioenergetics parameters in skeletal muscle of WT and ALS mice. Our data showed that the function of the electron transport chain (ETC) did not differ in the studied groups, but significant changes were observed in CS, MDH, and COX activities. Conversely, we showed that bioenergetics of mitochondria in skeletal muscle of *hSOD1 G93A* mice were significantly reduced in mice at the terminal stage of disease compared to WT and asymptomatic groups of mice. Decreased CS and elevated COX activities were also observed in skeletal muscle of the ALS mice at the terminal stage [36]. Tefera et al. demonstrated that, even at the first stage of the disease, the mRNA levels of other TCA enzymes, e.g., 2-oxoglutarate (OGDH) and succinate dehydrogenases, were significantly reduced but has no significant changes in OGDH activity [40]. Decreased activity of CS was noticed in muscle from ALS patients compared with healthy controls [41,42]. CS activity is generally used as a marker of mitochondrial content [43], but activity of this enzyme is limited by the availability of oxaloacetate (OAA) [40]. Reduced glucose metabolism in skeletal muscle of *hSOD1 G93A* mice, reported by Charbonnier [26], may help understand the changes in enzymatic activity. In ALS, reduced CS activity may be related to impaired glucose metabolism and decreased availability of OAA, which may not necessarily reflect the true amount of mitochondrial content in the muscles.

Other parameters describing mitochondrial bioenergetic efficiency and oxidative capacity, OXPHOS coupling efficiency (OCE), and MDH and COX activity were not in the line with changes in CS activity in the skeletal muscle of ALS mice. The lack of changes in OCE suggests that the ETC system is still coupled and can function, owning to the alternative process of proton supplying to the respiratory chain at the symptomatic stage of disease. Hydrogen can be alternatively sourced from amino acids (glutamate, isoleucine, and valine) [40]; however, the concentration of amino acids was not measured in this study. OCE (1−oxygen consumption before ADP and after ADP addition) is an indirect measurement of the ADP/ATP ratio [44]. Sustained function of mitochondrial respiration is in agreement with COX activity. We showed that significant elevation of the enzyme activity was also seen at the terminal stage of disease, as well as by increased protein content of COX subunits [36]. To the best of our knowledge, there are no data showing COX activity in mouse skeletal muscle in the *hSOD1 G93A* model compared to the control group. However, in skeletal muscle in patients with ALS, lower values of this enzyme activity were recorded [41,42]. These discrepancies may be due to the differences between the animal model of the disease and patients with ALS. The ratio of COX to CS activity at 0.5 to 0.7 AU in ALS groups corresponds to that described by Patel et al. [45] and confirms the discrepancy in the directions of changes for the activity of both oxygen metabolism enzymes following ALS.

As mentioned above, CS activity is limited by the availability of OAA [40]. To check whether under the condition of disturbed glucose metabolism the compensatory delivery of this metabolite from malate occurs, and whether further steps of the TCA cycle are maintained, we measured the activity of MDH. The increased activity of MDH isoenzymes in skeletal muscle of ALS mice compared with WT was observed. Notably, an increase in the activity of MDH and COX in the skeletal muscle of ALS mice was accompanied by a decrease in CS activity. According to Tefera et al. (2016) [40], maintaining the ATP production capacity by mitochondria appears to be neuroprotective and delays muscle wasting. The compensatory mechanism resulting in an increase in MDH activity seems to maintain the production of OAA in the muscles, and thus the Krebs cycle. This, in turn, supplies hydrogen to the respiratory chain and for the production of ATP. The lack of changes in OXPHOS coupling efficiency seen in our experiments confirms the potential compensation mechanism in mitochondrial bioenergetics.

Mitochondria, in addition to the bioenergetic function, under pathological conditions are also considered as the main cellular source of free radicals generation and oxidative stress [28,29,30]. The presence of the oxidative stress in ALS has been repeatedly reported [10,30]. In the present study, we also documented the decreased concentration of SH groups in ALS mice at the symptomatic stage of the disease, supporting the similar observation obtained in the skeletal muscle of ALS animals during the progression of disease [10,11,22,36]. We found a significant negative correlation between COX and MDH activities and SH group level in the ALS groups. Our data may suggest that increased oxygen energy metabolism in ALS animals is accompanied by increased ROS generation and elevated oxidative stress in the skeletal muscle of *hSOD1 G93A* mice.

Tefera et al. suggested that TCA in skeletal muscle might be slowed in *hSOD1 G93A* mice with concomitant decrease in muscle strength [40]. As such, we ascertained which of the markers of mitochondrial bioenergetics are correlated with changes in skeletal muscle strength and whether swim training influences these parameters at the symptomatic stage of the disease.

### 3.2. Effect of Swim Training on Changes in Muscle Strength, Oxidative Stress, and Energy Metabolism in Symptomatic ALS Mice

Swim training increases the lifespan of *hSOD1 G93A* mice by about 10%–13% [15,36]. From a clinical point of view, not only prolongation of lifespan, but also sustained functionality and inhibition of muscle waste are critical elements of therapy. In agreement with previously published data, swim training significantly decreases the reduction in muscle strength clearly visible at the symptomatic stage of ALS [15]. Swim training is characterized by non-weight bearing exercises that minimize damage to muscle fibers [46], reduces oxidative stress, and improves muscle energy metabolism at terminal stage of the disease [36]. However, swim training had no significant effect on oxidative stress or MDH and COX activity, and had no influence on oxygen consumption by mitochondrial respiratory chain at the symptomatic stage of the disease. Conversely, CS activity in skeletal muscle of ALS mice was significantly higher as a result of swim training and was significantly positively correlated with the muscle strength test. There are no data showing the effect of swim training on COX activity in the skeletal muscle of ALS mice.

## 4. Materials and Methods

### 4.1. Animals

The experiments with animals (project No. 2013/09/NZ7/02538) were approved by the Local Ethics Committee (Resolution No. 11/2013 of 22 April 2013) and in accordance with the approval of the Polish Ministry of the Environment (Decision No. 155/2012).

Transgenic male mice with the G93A human SOD1 mutation B6SJL-Tg (SOD1^G93A^) 1Gur/J (ALS mice) (*n* = 24) and wild-type male mice B6SJL (*n* = 24) were purchased from The Jackson Laboratory (Bar Harbor, ME, USA). The mice were housed in an environmentally controlled room (23 ± 1 °C with a 12 h light/dark cycle); the mice received standard mice chow and water ad libitum. After acclimatization, the mice were randomly divided into the following groups according to disease progression and training status: ALS 0: ALS untrained mice with no visible signs of the disease (*n* = 8), ALS I: ALS untrained (*n* = 8), and ALS I SWIM: and ALS trained mice (*n* = 8). Corresponding groups of wild-type (WT) untrained and trained mice were created: WT 0: WT untrained mice (*n* = 8), WT I: WT untrained mice (*n* = 8), and WT I SWIM: WT trained mice (*n* = 8).

The mice were euthanized by cervical dislocation. The mice from the ALS 0 and WT 0 groups were euthanized on the 70th day of life. The mice from the ALS I group were euthanized when we observed the first symptoms of disease (116 ± 2 days of life). The ALS I SWIM, WT I, and WT I SWIM mice were euthanized at the same age as the ALS I group.

### 4.2. Swim Training Protocol

Starting at 10 weeks of age, the transgenic (group ALS I SWIM) and the control mice (group WT I SWIM) underwent the training procedure according to Deforges et al. [15] with slight modification described by Flis et al. [36].

### 4.3. Grip Strength

Grip strength (*n* = 8 in each group) was tested once a week between 70 and 105 days of life. The time during which the animals were able to sustain their weight holding onto a metal rail suspended midair was recorded, with the maximum time being 180 s. Each mouse was subjected to three trials with a resting period between tests.

### 4.4. Isolation of Skeletal Muscle Mitochondria

The thigh muscle mitochondria were isolated, as previously described by Makinen and Lee [47] with slight modifications described by Flis et al. [36]. The muscles were rapidly removed, trimmed of visible connective tissue, weighed, and placed in 10 mL of ice-cold mitochondrial isolation buffer A (100 mM KCl, 50 mM Tris, 5 mM MgCl_2_, 5 mM EDTA, pH 7.4). The muscles were minced with scissors, incubated for 1 min with protease (10 mL of isolation buffer per 1 g of tissue, supplemented with protease (0.2 mg/mL)). After 1 min. of incubation, the same volume of buffer A was added and homogenized using a Teflon pestle homogenizer. The homogenate was centrifuged at 700× *g* for 10 min. The supernatant was decanted and centrifuged at 4 000× *g* for 10 min. The mitochondrial pellet was resuspended in 30 mL of suspension buffer B (100 mM KCl, 50 mM Tris, 1 mM MgCl_2_, 1 mM EDTA, pH 7.4, supplemented with 0.5 % BSA) and centrifuged at 10,000× *g* for 10 min. After this washing step had been repeated twice, the final mitochondrial pellet was resuspended in buffer MRB (250 mM mannitol, 5 mM HEPES, 0.5 mM EGTA, pH 7.4) (skeletal muscle mass (mg) × 0.2 mL). All the steps were performed at 4 °C.

### 4.5. High-Resolution Respirometry

Respiration was measured at 37 °C in a high-resolution respirometer (Oroboros, Oxygraph; Innsbruck, Austria) according to [36]. Freshly isolated skeletal muscle mitochondria (0.1 mg of mitochondrial protein) were added to 2 mL of the respiration medium (110 mM sucrose, 60 mM K-lactobionate, 0.5 mM EGTA, 0.1 % BSA essentially fatty acid free, 3 mM MgCl_2_, 20 mM taurine, 10 mM KH_2_PO_4_, 20 mM K-HEPES, pH 7.1). For the assessment of mitochondrial respiration, the following protocol was used: (1) Non-phosphorylating LEAK respiration was assessed by injecting 2 mM L-malic acid (neutralized with KOH) as NADH (N)-linked substrates, called state N*L*; (2) OXPHOS capacity was induced by adding 5 mM ADP and 10 mM of glutamic acid (neutralized with KOH), called state N*P*; and (3) OXPHOS coupling efficiency (OCE), calculated with the formula (1 − (state N*L*)/(state N*P*)), reflects the coupling of respiration supported by electron transferring flavoprotein (ETF) with malate as substrate before (state N*L*) and after the addition of ADP and glutamic acid (state N*P*). The OCE was calculated as a measure of mitochondrial quality and control.

### 4.6. Measurement of Cytochrome c Oxidase, Citrate Synthase and Malate Dehydrogenase Activities

All enzymes activities were measured spectrophotometrically (Cecil CE9200, Cecil Instruments Limited, Cambridge, UK) in thigh muscles homogenates. The enzymes activities are expressed as μmol/minute/mg of protein. The cytochrome c oxidase (COX) activity was measured at 37 °C, according to Reference [48]. Briefly, 20 μL of homogenate (1:10, 5%) was incubated for 2 min in 960 μL of buffer (10 mM potassium phosphate buffer, 0.01% Triton-X100, pH 7.2). Next 20 μL of reduced cytochrome *c* was added to initiate the reaction. The reactions were conducted in duplicate and absorbance was read at 550 nm.

The citrate synthase (CS) activity was measured at 37 °h in duplicate according to Reference [49]. Briefly, 10 μL of homogenate (1:10, 5%) was incubated for 2 min in 970 μL of buffer (50 mM Tris-HCl, 1 mM EDTA, 0.01% Triton-X100, pH 7.8) supplemented with 10 μL of freshly made DTNB (10 mM) and 10 μL acetylCoA (50 mM). Next 10 μL of freshly made oxaloacetic acid (10 mM) was added to initiate the reaction. The reactions were conducted in duplicate and absorbance was read at 412 nm.

The malate dehydrogenase (MDH) activity was measured at 30 °C according to [50]. Briefly, 10 μL of homogenate (1:10, 5%) was incubated for 2 min in 970 μL of buffer (50 mM Tris-HCl, 5 mM EDTA, 0.01% Triton-X100, pH 7.6) supplemented with 10 μL of freshly made NADH (20 mM). Next 8.5 μL of freshly made oxaloacetic acid (20 mM) was added to initiate the reaction. The reactions were conducted in duplicate and absorbance was read at 340 nm. The mitochondrial MDH (mMDH) activity was assessed after 2 min preincubation of 20 μL homogenate (1:10, 5%) with 98% ethanol (1:1 vol). The cytosolic MDH (cMDH) was calculated as the difference between MDH and mMDH activities.

### 4.7. Manifestation of Oxidative Stress

The sulfhydryl groups (SH) [51] were measured in the skeletal muscles homogenates. Briefly, 20 μL of homogenate (5%) was added to 200 μL of buffer (10 mM potassium phosphate buffer, pH 8.0). Next 30 μL of SDS (10 %) and 30 μL of DTNB (1 mM) were added. The measurements were conducted in duplicate. After this, the probes were mixed and incubated for 30 min in 37 °C. The absorbance was measured at 412 nm in microplate reader Thermo Scientific Multiscan Go (ThermoFisher Scientific, Vartaa, Finland). The values of the sulfhydryl groups (mmol/g of tissue) were calculated against sample blank (without DTNB) from the standard curve of reduced glutathione.

### 4.8. Data Analysis

Statistical analyses were performed using a software package Statistica v. 13.0 (StatSoft Inc., Tulsa, OK, USA). The results are expressed as the mean ± standard error (SE). The differences between the group means for tissue-based assays were performed using two-way ANOVA. The changes in mean scores of grip strength over time points were tested using the repeated measures ANOVA. If a difference was detected in the ANOVA model, the significant differences were determined using the Tukey’s post-hoc test. To verify the significance of the small, swim training associated changes (ALS TER vs. ALS SWIM), the Least Significant Difference (LSD) post-hoc test was used. The results were considered statistically significant when *p* < 0.05. A Pearson product-moment correlation coefficient was computed to assess the relationship between obtained results.

## 5. Conclusions

Amyothropic lateral sclerosis disease significantly reduces the muscle strength even at the early stage of the disease. ALS is also related to increased oxidative stress and modulation of muscle metabolism. Swim training significantly slows the reduction in muscle strength and increases CS activity in skeletal muscle. Our findings indicate that swim training is a modulator of skeletal muscle energy metabolism with concomitant improvement of skeletal muscle function in ALS mice (Figure 6).

## Figures and Tables

**Table 1:** Mitochondrial bioenergetics in skeletal muscle of the ALS and WT mice. Non-phosphorylating LEAK respiration (state NL), OXPHOS capacity (state NP) and OXPHOS coupling efficiency (OCE), were measured in skeletal muscle mitochondria. There were no significant differences between the groups. The data are presented as the means ± SEM (*n* = 5 in each group).

	WT			ALS		
	0	I	I SWIM	0	I	I SWIM
state N*L*	27.86 ± 2.06	29.43 ± 1.18	30.06 ± 1.96	23.87 ± 1.78	28.96 ± 1.12	29.84 ± 1.16
state N*P*	410.1 ± 38.53	492.5 ± 19.78	434.6 ± 47.36	371.4 ± 41.26	424.2 ± 38.54	379.7 ± 29.95
OCE	0.930 ± 0.006	0.931 ± 0.007	0.933 ± 0.008	0.920 ± 0.013	0.920 ± 0.010	0.928 ± 0.005
